# Poststroke sleep phenotypes and executive dysfunction: toward a sleep-network-executive framework

**DOI:** 10.3389/fnins.2026.1978796

**Published:** 2026-10-21

**Authors:** Jincheng Xu, Yan Zhang, Dandan Lin, Xue Yan, Nanyun Xie, Sutao Si, Shuming Yang

**Affiliations:** 1 Department of Rehabilitation Medicine, Wuxi Huishan District People's Hospital, Wuxi, Jiangsu, China; 2 Department of Rehabilitation, The Affiliated Mental Health Center of Jiangnan University, Wuxi Mental Health Center, Wuxi, Jiangsu, China

**Keywords:** default mode network, executive dysfunction, frontoparietal network, poststroke cognitive impairment, poststroke sleep disturbance, salience network, sleep-network-executive framework

## Abstract

Post-stroke sleep disturbance and executive dysfunction frequently coexist, yet the mechanisms linking disturbed sleep to cognitive recovery remain poorly understood. A major challenge is phenotypic heterogeneity: insomnia, sleep fragmentation, obstructive sleep apnea, hypersomnolence, and circadian disruption differ in their biological drivers and cognitive consequences. To develop a testable network-level account, we conducted a targeted synthesis of clinical stroke studies, stroke neuroimaging research, non-stroke sleep-network evidence, and intervention studies, mapping each evidence stream according to its proximity to the proposed pathway. The synthesis indicates that focal lesions, diaschisis, and structural disconnection after stroke can disrupt the integrity and coordination of the salience, default mode, and frontoparietal networks. Clinical studies support an association between disturbed sleep and poorer post-stroke cognition, while neuroimaging findings demonstrate altered large scale network organization in post-stroke cognitive impairment. Evidence from non-stroke populations further suggests that sleep loss, insomnia, and sleep fragmentation can impair network segregation, default mode regulation, frontoparietal recruitment, and salience-network-mediated switching. We therefore propose that sleep fragmentation, insomnia, and arousal instability may act as functional stressors on an already vulnerable post-stroke network architecture. Inefficient switching may permit context-inappropriate persistence of default mode activity and reduce or delay frontoparietal engagement during executive demand, thereby contributing to impaired conflict monitoring, working memory, processing speed, and cognitive flexibility. No study has directly tested this complete pathway within a single stroke cohort. The framework should therefore be regarded as a falsifiable mechanistic hypothesis rather than an established causal model. Future studies should combine objective sleep phenotyping, dynamic and effective connectivity, task based neuroimaging, domain specific executive measures, and longitudinal or interventional designs within the same stroke cohorts.

## 1 Introduction

Stroke remains a major global cause of death and disability (GBD 2021 Stroke Risk Factor Collaborators, 2024). Sleep and cognitive disturbances are common after stroke and may interfere with rehabilitation. Meta-analyses indicate that poor sleep quality affects approximately half of stroke survivors, with pooled prevalence estimates of 50.3–53.0% (Luo et al., 2023; Wang et al., 2025). Poststroke cognitive impairment (PSCI) is similarly common, although prevalence estimates vary according to case definition, assessment method, study population, and time since stroke (Huang et al., 2022; Ma et al., 2025). Sleep and cognitive deficits may occur in the same individuals, but their joint prevalence has not been reliably quantified. Available evidence nevertheless suggests that greater sleep fragmentation and lower sleep efficiency are associated with poorer cognitive performance after stroke (Falck et al., 2019; Ihle-Hansen et al., 2025). This overlap is clinically important: disrupted sleep can reduce rehabilitation tolerance and day to day engagement (Fulk et al., 2020), whereas executive dysfunction is associated with slower functional gains and poorer reintegration into the community (Zhou et al., 2025). How poststroke sleep disturbance relates to cognitive dysfunction is still not fully understood. Existing studies generally show that poor sleep is associated with worse cognitive outcomes after stroke, but they do not clearly establish whether sleep disturbance contributes to cognitive decline, reflects a parallel consequence of brain injury, or interacts with other poststroke processes, including inflammation, circadian disruption, mood symptoms, and reduced daytime activity (Chen et al., 2024; Zhang et al., 2026; Pawluk et al., 2025). Interpretation is further complicated by heterogeneity in both constructs. Sleep disturbance may refer to insomnia, sleep fragmentation, sleep disordered breathing, hypersomnolence, or circadian dysregulation, whereas cognitive impairment includes multiple domains with distinct neurobiological substrates. These distinctions are particularly relevant for executive dysfunction, which relies on coordinated activity across large scale networks and has major implications for rehabilitation participation and adaptive daily functioning.

A purely lesion centered account is unlikely to explain this complexity. Stroke is increasingly viewed as a disorder that disrupts distributed brain communication, rather than as a solely focal event (Siegel et al., 2016; Grefkes and Fink, 2014; Wang et al., 2024). In this context, the triple network model is useful because it focuses on interactions among the salience network (SN), default mode network (DMN), and frontoparietal network (FPN) (Wang et al., 2024; Menon, 2011). The SN is thought to support switching between internally and externally oriented processing states (Seeley et al., 2007; Sridharan et al., 2008), while the DMN and FPN contribute primarily to internally directed cognition and executive control, respectively (Buckner and DiNicola, 2019; Vincent et al., 2008). Coordination among these systems is central to attentional control, conflict monitoring, and working memory, all of which are frequently affected after stroke. Sleep disruption may be especially relevant to this network organization. In non stroke populations, sleep fragmentation, insomnia, and acute sleep loss alter large scale functional architecture, including network segregation, global efficiency, and the balance between default mode and control related activity (De Havas et al., 2012; Chee and Zhou, 2019; Ben Simon et al., 2017). Evidence also points to a role for the SN in reconfiguring networks as cognitive demands change (Sridharan et al., 2008; Goulden et al., 2014). These findings raise the possibility that poststroke sleep disturbance may further destabilize an already vulnerable system by weakening SN mediated switching, reducing appropriate DMN suppression, and limiting FPN recruitment during cognitively demanding states. Such effects would be expected to appear most clearly in executive processes such as conflict monitoring and working memory, which can be examined using paradigms such as the Stroop and N-back tasks (Periáñez et al., 2021; Owen et al., 2005).

To examine this possibility, we conducted a targeted narrative synthesis rather than a quantitative meta analysis, with the aim of developing a mechanistic framework that could be tested in future stroke cohorts. The central contribution of this review is not to summarize associations between sleep and cognition after stroke, but to propose a falsifiable network level mechanism through which selected sleep phenotypes may amplify executive vulnerability in the poststroke brain. Accordingly, the review integrates clinical, sleep, neuroimaging, and intervention related evidence relevant to the proposed sleep network executive pathway. Building on recent advances in dynamic and effective connectivity, complemented by task based functional magnetic resonance imaging (fMRI) and psychophysiological interaction analyses, we examine whether poststroke sleep disturbance may contribute to executive dysfunction by impairing salience network mediated switching between default mode and frontoparietal systems (Cohen, 2018; Hutchison et al., 2013; Ruan et al., 2025; Friston et al., 1997; Elton and Gao, 2014). We first synthesize clinical evidence on the overlap between sleep disturbance and cognitive impairment after stroke, then review triple network abnormalities in PSCI and sleep related perturbations of network dynamics. On this basis, we propose a testable Sleep-network-executive framework intended to support research stratification, guide mechanism sensitive outcome selection, and inform future intervention studies.

## 2 Literature search and evidence mapping

Relevant literature was identified through searches of PubMed, Web of Science, and Scopus, supplemented by Google Scholar and manual screening of reference lists from key reviews and primary studies. Searches covered literature available up to March 20, 2026. Search keys combined stroke related terms, sleep phenotype terms, cognitive and executive function terms, and network level terms including default mode network, frontoparietal network, salience network, functional connectivity, dynamic functional connectivity, and effective connectivity. Full database specific search strategies are provided in Supplementary Table S1.

Eligible sources included peer-reviewed original empirical studies and relevant reviews. Original studies were prioritized when they addressed any of the following topics:

- Poststroke sleep phenotypes and cognitive outcomes;
- Large-scale network abnormalities in poststroke cognitive impairment;
- Stroke-specific sleep-related neuroimaging findings;
- Sleep-related alterations in network dynamics; or
- Sleep, respiratory, circadian, behavioral, and neuromodulatory interventions relevant to executive recovery.

Reviews and systematic reviews were used to contextualize the literature, identify convergent themes and evidence gaps, and support conceptual integration rather than as substitutes for primary evidence. Experimental sleep-deprivation studies in non-stroke populations were retained only when they informed network-level mechanisms relevant to the proposed framework. Studies were excluded if they lacked clear relevance to stroke, sleep, cognition, or large-scale network function, or if they did not provide empirical, methodological, or conceptual evidence relevant to any prespecified evidence tier. Studies were not excluded because their findings were negative, inconsistent, or contrary to the proposed framework. Evidence was mapped according to prespecified tiers based on proximity to the proposed sleep–network–executive pathway, including direct clinical evidence, stroke-specific network evidence, preliminary bridge evidence, indirect mechanistic evidence, and translational evidence. Evidence-tier definitions and mapping criteria are provided in Supplementary Table S2. When evidence was inconsistent, interpretation emphasized study design, sleep phenotype specificity, objective versus subjective sleep measurement, cognitive domain specificity, imaging approach, and stroke stage or lesion/disconnection profile. No formal risk of bias assessment or quantitative synthesis was performed. The absence of direct validation of the complete sleep SN-DMN-FPN executive pathway within a single poststroke cohort was treated as a central evidence gap rather than as established mechanistic evidence. Transparency of the narrative synthesis was self audited using SANRA criteria, with details provided in Supplementary Table S3.

## 3 Clinical and conceptual background

Clarifying the relationship between PSSD and cognitive impairment requires more precise characterization of both sleep and cognitive phenotypes, together with an approach that goes beyond classical lesion centered neuroanatomy. Although PSSD is highly prevalent, it is often treated as a single clinical entity despite substantial heterogeneity. In practice, it includes several partially overlapping phenotypes, particularly obstructive sleep apnea (OSA), insomnia, sleep fragmentation, and circadian misalignment, each of which may involve different pathophysiological mechanisms (Rissardo et al., 2025). OSA is characterized by intermittent hypoxia and recurrent micro arousals and may contribute to neuroinflammation, oxidative stress, and vascular dysfunction (Lv et al., 2023). By contrast, poststroke insomnia and sleep fragmentation are more closely linked to hyperarousal, unstable sleep continuity, and disruption of restorative sleep processes (Pérez-Carbonell and Bashir, 2020; Mayer et al., 2011). Treating these phenotypes as mechanistically equivalent may obscure important differences in how sleep disturbance affects neuroplasticity, arousal regulation, and large scale network organization after stroke.

Similar precision is needed for cognitive outcomes. PSCI spans memory, language, visuospatial function, attention, and executive control (Huang et al., 2022). Executive dysfunction deserves particular attention because it is common after stroke, strongly associated with functional dependence, and directly relevant to rehabilitation participation. Working memory, cognitive flexibility, and conflict monitoring are central to goal directed behavior (Friedman and Miyake, 2017), but these capacities depend less on isolated cortical regions than on rapid, coordinated interactions across distributed systems. This dependence makes executive function vulnerable not only to structural disconnection, but also to state dependent changes in arousal and network dynamics associated with poor sleep (Pesoli et al., 2022).

Clinical observations are consistent with a link between PSSD and adverse cognitive outcomes, particularly in attention and executive domains (Li et al., 2019). Patients with poor poststroke sleep quality or persistent sleep disturbance often perform worse in attention and executive related tasks than those with more stable sleep (Li et al., 2019; Kaneko et al., 2003). These associations are clinically meaningful because sleep related cognitive difficulties may also reduce rehabilitation tolerance and slow functional recovery (Li et al., 2019). However, the evidence remains methodologically constrained. Many studies rely on subjective sleep questionnaires and global cognitive screening tools, which may not adequately capture temporal sleep instability, sleep microstructure, or domain specific executive deficits. Causal direction is also uncertain, since cognitive impairment, depression, reduced daytime activity, and medication effects may themselves worsen sleep (Chen et al., 2024). Further progress will require integrating objective sleep measures, such as polysomnography (PSG) or actigraphy, with sensitive assessments of executive subdomains and longitudinal recovery trajectories.

These considerations also help explain why lesion based accounts, although important, are insufficient on their own. The co-occurrence of sleep and cognitive deficits after stroke has often been interpreted in relation to focal injury involving shared neuroanatomical substrates, such as the thalamus or prefrontal cortex, or to broader secondary processes such as inflammation (Pawluk et al., 2025; Mayer et al., 2011; Filchenko et al., 2023). These explanations remain relevant, but they do not fully account for the marked heterogeneity in poststroke sleep and cognitive trajectories. Patients with apparently similar lesion topography may show very different patterns of sleep disruption, executive impairment, and recovery. One likely reason is that stroke affects more than the lesion site itself, involving diaschisis, disruption of long range white matter pathways, and altered communication across distributed cortical and subcortical systems (Carrera and Tononi, 2014; Salvalaggio et al., 2020; Koh et al., 2021). Because executive dysfunction depends on flexible coordination among large scale control systems (Cole et al., 2013; Dosenbach et al., 2008), the combined burden of PSSD and PSCI is better understood at the network level than through lesion location alone. These observations motivate a shift from broad symptom labels and lesion centered explanations toward a network level account of poststroke sleep–cognition interactions. Figure 1 provides the clinical and conceptual rationale for this shift, showing how the overlap between PSSD and PSCI, the heterogeneity of both sleep and cognitive phenotypes, and the limitations of conventional explanatory models converge on the need for a sleep–network–executive framework.

**Figure 1:**
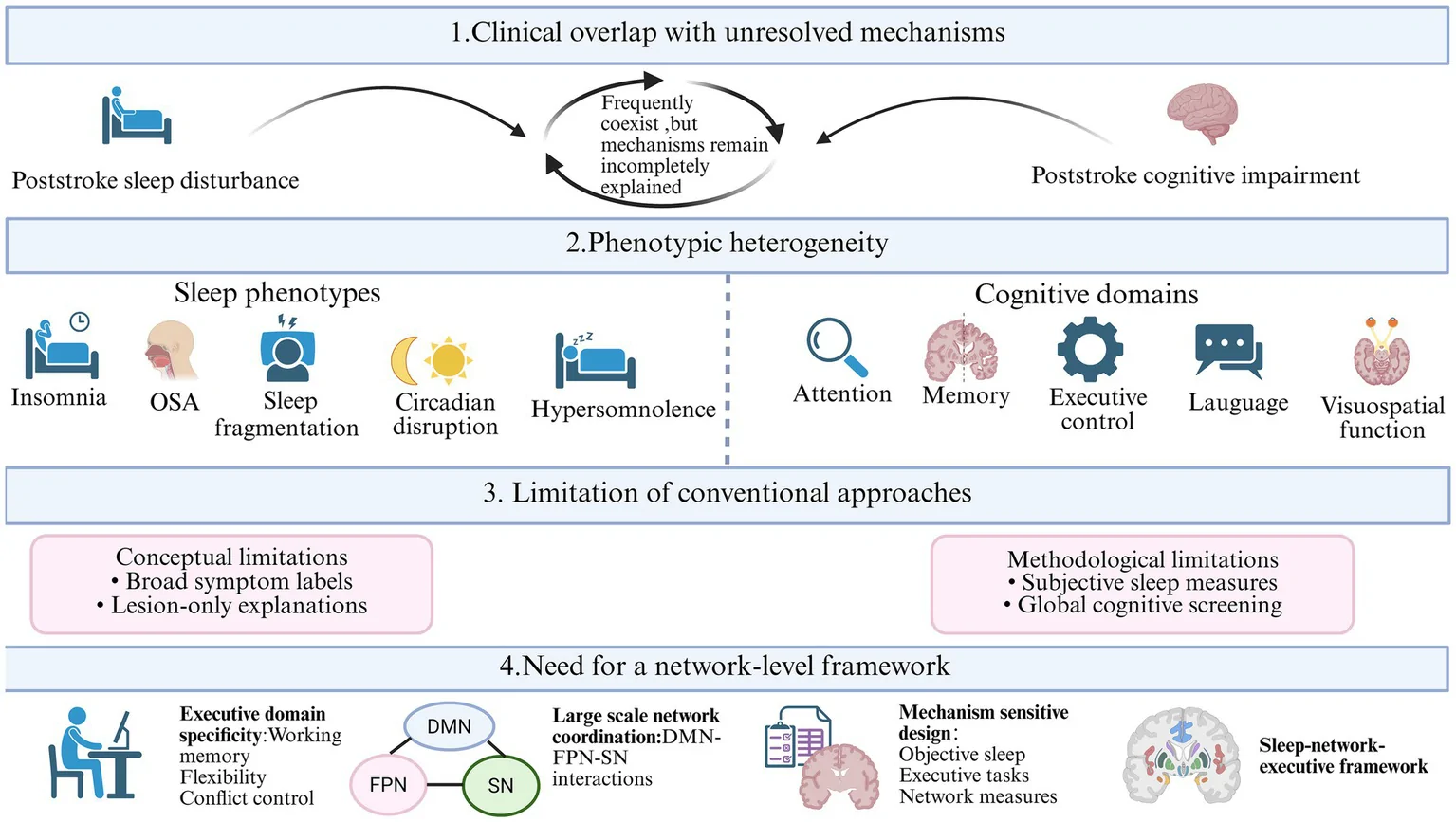
Clinical and conceptual rationale for a sleep-network-executive framework after stroke. Poststroke sleep disturbance and cognitive impairment frequently overlap but remain mechanistically heterogeneous. The figure highlights why broad symptom labels and lesion centered accounts are insufficient, motivating a network level framework focused on executive dysfunction.

## 4 Triple network abnormalities in poststroke cognitive impairment: relevance to sleep related executive dysfunction

Accumulating evidence suggests that PSCI reflects not only focal tissue injury, but also disrupted coordination across large-scale brain networks (Ruan et al., 2025; Yue et al., 2023). The triple network model is useful in this context because it relates internally directed cognition, executive control, and salience-mediated switching. After stroke, altered interactions among the DMN, FPN, and SN may create a vulnerability to additional disturbances, including poor sleep (Wang et al., 2024; Menon, 2011).

The DMN supports self-referential processing and internally oriented cognition. During externally directed executive tasks, appropriate reduction of DMN activity helps limit interference from task-unrelated processing (Buckner and DiNicola, 2019; Anticevic et al., 2012). In PSCI, neuroimaging studies have reported reduced within-network coherence and abnormal interactions between the DMN and other systems (Wang et al., 2024; Yue et al., 2023; Sagues et al., 2025). The FPN contributes to working memory, cognitive flexibility, task-set maintenance, and top-down behavioral regulation (Vincent et al., 2008; Cole et al., 2013; Dosenbach et al., 2008). Its involvement in PSCI needs to be considered alongside structural disconnection affecting other regions involved in cognitive control. For example, disconnectome mapping in 102 stroke survivors linked disconnection involving the right insula and frontal operculum, superior temporal gyrus, and putamen to poorer cognitive performance (Kolskår et al., 2022).

The SN is thought to support detection of behaviorally relevant information and transitions between internally and externally oriented processing states (Seeley et al., 2007; Sridharan et al., 2008; Goulden et al., 2014; Schimmelpfennig et al., 2023; Uddin, 2015). Within the triple network model, SN dysfunction may interfere with timely DMN disengagement and recruitment of frontoparietal control systems (Menon, 2011; Sridharan et al., 2008; Goulden et al., 2014; Uddin, 2015). Stroke lesion evidence supports the relevance of the insula to executive control: Varjačić et al. found that left insular lesions were associated with poorer set-switching in 144 patients with acute stroke. The association remained after accounting for the visuospatial and motor demands of the task (Varjačić et al., 2018). This finding supports an insular contribution to flexible behavior, although it does not directly measure switching between large-scale networks.

Studies combining static and dynamic analyses show why connectivity strength alone may be insufficient to characterize this dysfunction. Wang et al. examined 118 patients with left or right internal capsule stroke and 57 healthy controls. Both stroke groups showed increased static SN–DMN connectivity but reduced dynamic interactions between the executive control network and other networks. Patients with right-sided lesions also showed reduced modularity and greater persistence of a state with strongly positive connections across the three networks (Wang et al., 2024). Increased coupling can therefore coexist with less differentiated network organization.

Dynamic functional connectivity extends static analyses by describing the time spent in recurring network states and transitions between them (Cohen, 2018; Hutchison et al., 2013; Yue et al., 2023; Bonkhoff et al., 2021a, 2021b). In 17 patients with PSCI and 24 controls, Yue et al. found reduced static connectivity between the ventral DMN and SN. Patients also spent longer in one recurring connectivity state, and longer dwell time was associated with lower Mini-Mental State Examination scores (Yue et al., 2023). The cognitive association concerned a global screening score, leaving its relationship with specific executive processes uncertain.

Effective connectivity adds information about the direction of influence between regions. Using spectral dynamic causal modelling, Ruan et al. compared 41 patients with PSCI and 41 controls. Patients showed increased effective connectivity from the posterior cingulate cortex to bilateral dorsolateral prefrontal regions and from the right insula to the right dorsolateral prefrontal cortex. Greater connectivity was associated with poorer cognitive scores (Ruan et al., 2025). These results caution against equating stronger connectivity with better function. They support altered network coordination after stroke, while the contribution of sleep disturbance to these changes remains to be established.

## 5 Sleep related perturbation of large scale network dynamics

Sleep research adds a complementary line of evidence. In non-stroke populations, sleep loss can impair cognitive performance and alter coordination across large-scale brain networks (De Havas et al., 2012; Chee and Zhou, 2019; Pesoli et al., 2022; Killgore, 2010). These findings help identify processes that may be vulnerable after stroke, although experimental sleep deprivation and chronic clinical sleep disorders represent different exposures.

Restorative sleep is thought to support synaptic homeostasis and network recalibration (Tononi and Cirelli, 2014). Studies of sleep deprivation and restriction have reported changes in modular organization, network topology, and connectivity involving default mode and attention systems (Ben Simon et al., 2017; Xu et al., 2023; Zhang et al., 2025; Farahani et al., 2019). De Havas et al. scanned 26 healthy participants after normal sleep and approximately 24 h of total sleep deprivation. Sleep deprivation reduced connectivity within the DMN and weakened its anticorrelation with task-positive regions during both rest and a visual attention task (De Havas et al., 2012). Dai et al. studied 22 healthy participants before and after 36 h of sleep deprivation. Working memory performance declined, accompanied by increased connectivity between dorsal attention and default mode regions and decreased connectivity between frontoparietal and default mode regions (Dai et al., 2020). These observations connect experimental sleep loss with measurable changes in both cognition and network relationships.

The SN may be relevant because of its role in salience detection and network switching (Sridharan et al., 2008; Goulden et al., 2014; Uddin, 2015). Sleep-deprivation research has linked altered insula–prefrontal connectivity to reduced vigilant attention (Qi et al., 2021). In insomnia, however, abnormalities may be more apparent in temporal variability than in average connectivity. Wei et al. compared 65 people with insomnia disorder and 65 matched controls. Average between-network connectivity did not differ significantly after multiple-comparison correction, whereas connectivity between the anterior SN and left executive control network varied less over time (Wei et al., 2020). This finding is consistent with less flexible interactions at rest, but does not directly establish impaired switching during executive tasks.

Sleep continuity also warrants separate consideration. Observational studies have associated better continuity with better inhibitory control and other cognitive outcomes, with differences across age groups and executive domains (Wilckens et al., 2014). In older adults, Boeve et al. found that sleep efficiency was associated with response inhibition, whereas daytime sleepiness or fatigue was associated with cognitive flexibility; subjective sleep quality was not significantly associated with executive performance (Boeve et al., 2022). Experimental findings are less uniform. Ferri et al. exposed 15 healthy adults to two nights of sleep fragmentation. Despite reduced slow-wave sleep and altered sleep microstructure, cognitive performance did not generally deteriorate, and some mental rotation measures improved (Ferri et al., 2010). The effects of fragmented sleep may therefore depend on exposure duration, the cognitive task, and the capacity to tolerate disrupted sleep.

OSA involves additional respiratory and hypoxic influences. Prilipko et al. used a visuospatial N-back task in 17 men with untreated moderate-to-severe OSA and seven controls. Patients showed altered task-positive recruitment and DMN responses across task loads. Nocturnal desaturation was associated with activity in both networks, while higher body mass index was associated with poorer task performance (Prilipko et al., 2011). OSA related findings should therefore not be attributed to sleep fragmentation alone. For the proposed framework, the question is which features of disturbed sleep affect network coordination, and whether pre-existing stroke-related injury increases their cognitive consequences.

## 6 Stroke specific evidence and implications

Stroke-specific evidence linking sleep disturbance to large-scale network dysfunction remains limited. Clinical and imaging findings nevertheless suggest that poststroke sleep disturbance cannot be understood solely from damage to individual sleep-regulating structures (Baillieul et al., 2023; Cai et al., 2021). The available studies address different parts of the proposed pathway, with important differences between insomnia, disrupted sleep continuity, and sleep-disordered breathing.

Studies of poststroke insomnia provide an initial imaging basis. Wang et al. recruited 27 patients at least six months after ischemic stroke; 23 remained after imaging quality control. They assessed sleep using the Pittsburgh Sleep Quality Index (PSQI) and analyzed resting-state fMRI with regional homogeneity (ReHo), amplitude of low-frequency fluctuations (ALFF), and fractional ALFF. Compared with healthy controls, patients showed elevated ALFF in the right precuneus and left posterior cingulate gyrus, among other regions. ReHo was lower in bilateral lingual and right sensorimotor regions but higher in the left supramarginal gyrus, where it correlated negatively with PSQI scores (Wang et al., 2022). These abnormalities involved cortical regions beyond the subcortical infarcts, including parts of the DMN. However, ALFF and ReHo describe local activity and synchrony; they do not measure SN–DMN–FPN switching. Elevated resting activity cannot establish insufficient DMN suppression during executive demand. Without a stroke group free of insomnia, the study also cannot separate stroke-related changes from those specifically associated with insomnia (Wang et al., 2022). The interpretation of these findings as a possible hyperarousal pattern draws partly on non-stroke insomnia research. That literature implicates default mode activity, sensory processing, and affective arousal, but reports variable regional and connectivity findings (Marques et al., 2018; Khazaie et al., 2017; Schiel et al., 2020). It provides a basis for asking whether persistent internal activity or heightened responsiveness to stimuli interferes with executive control after stroke. Such interference was not directly tested in the poststroke insomnia imaging study.

Clinical studies offer additional evidence on sleep continuity. Falck et al. compared 35 older adults with chronic stroke and 35 matched adults without stroke using actigraphy, the PSQI, and the ADAS-Cog Plus. Participants with stroke had greater actigraphic sleep fragmentation despite similar PSQI scores. Cognitive differences between groups were larger in the presence of greater fragmentation or lower sleep efficiency (Falck et al., 2019). The discrepancy between objective and subjective sleep measures is relevant when defining poststroke sleep phenotypes. However, the cross-sectional design and composite cognitive outcome do not establish whether disrupted sleep preceded cognitive difficulties or preferentially affected executive function.

Respiratory disturbance introduces a different set of considerations. Li et al. assessed 86 patients within 14 days of minor ischemic stroke using polysomnography and cognitive testing. Attention, working memory, and executive function were frequently impaired. Patients with PSCI had a higher prevalence of OSA and shorter total sleep time than those without PSCI. Total sleep time and minimum nocturnal oxygen saturation remained associated with PSCI in regression analyses (Li et al., 2019). These results support separate assessment of sleep duration and hypoxic burden, although they do not identify the network processes underlying the cognitive associations.

Considered alongside the network abnormalities described in PSCI, these findings motivate the hypothesis that disturbed sleep places additional demands on an already compromised system (Sagues et al., 2025; Wei et al., 2020; Wang et al., 2022). Reduced SN-mediated switching could contribute to insufficient DMN suppression or delayed FPN recruitment during executive demand (Sridharan et al., 2008; Goulden et al., 2014; Dosenbach et al., 2008; Wei et al., 2020). This proposed sequence remains an inference across studies. Local abnormalities in poststroke insomnia, sleep–cognition associations, and altered network dynamics have largely been examined in separate samples.

Few stroke studies have jointly characterized detailed sleep phenotypes, network dynamics, and domain-specific executive performance. The evidence supports testing a link between these processes, but does not yet demonstrate the full pathway. Figure 2 maps the findings according to their proximity to the proposed sleep–network–executive mechanism. Table 1 summarizes the evidence layers and their limitations, distinguishing direct clinical associations from indirect mechanistic and preliminary stroke-specific imaging evidence.

**Figure 2:**
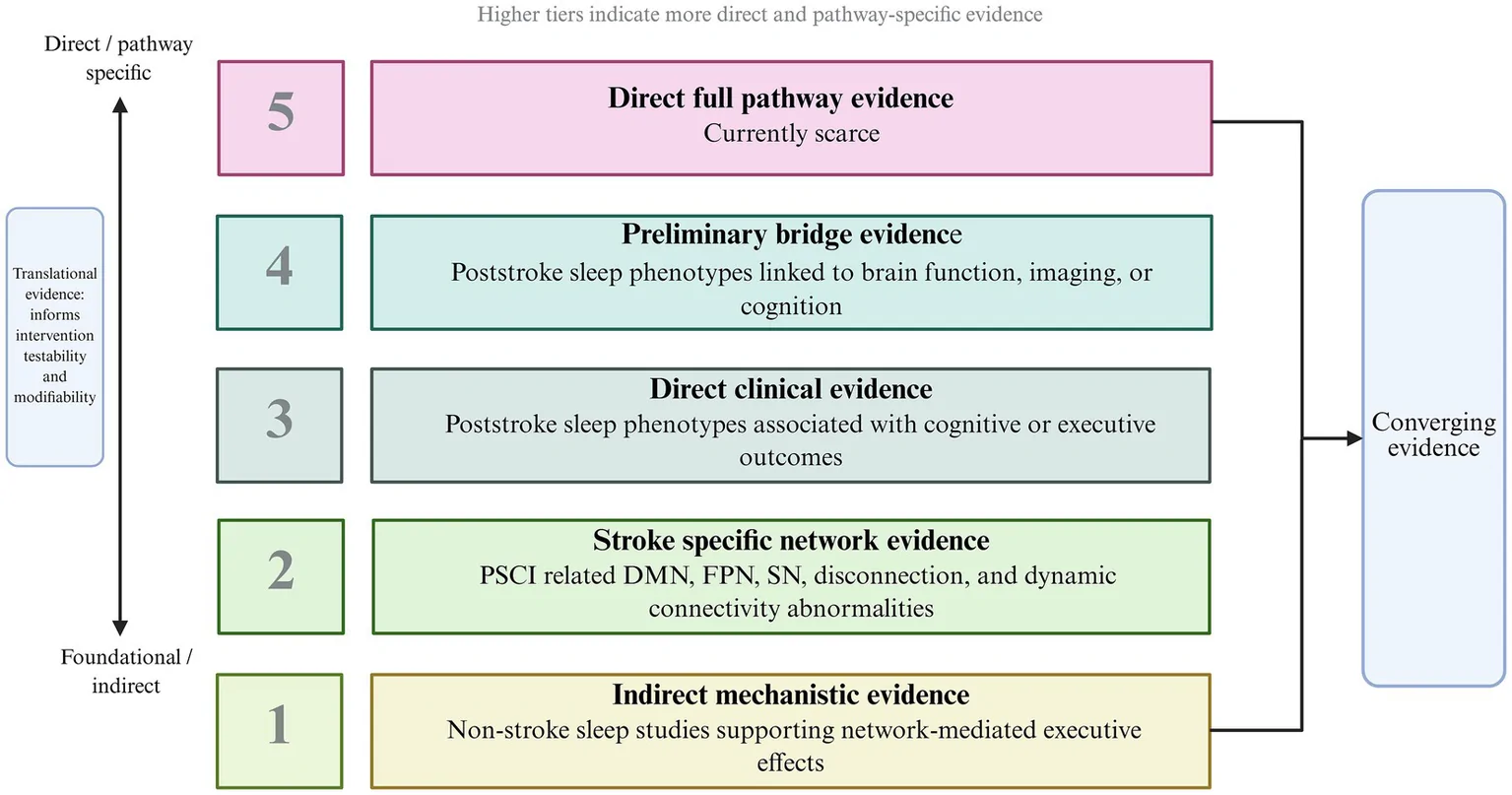
Evidence proximity map supporting the proposed sleep-network-executive framework. Evidence is organized from indirect mechanistic evidence to direct full pathway validation. Current evidence is convergent but incomplete, with no stroke cohort yet jointly testing sleep phenotypes, SN-DMN-FPN dynamics, and domain specific executive outcomes. Translational evidence informs intervention testability and pathway modifiability but is not shown as a separate proximity tier.

**Table 1:** Stratified evidence supporting the proposed sleep-network-executive framework after stroke.

Evidence domain	Representative references	Main contribution	Relevance to proposed framework	Evidence proximity category	Key limitations
Poststroke sleep disturbance and cognition	Luo et al. (2023), Wang et al. (2025), Huang et al. (2022), Ma et al. (2025), Falck et al. (2019), Ihle-Hansen et al. (2025), Fulk et al. (2020), Zhou et al. (2025), Li et al. (2019), Kaneko et al. (2003), and Zhang et al. (2023)	Clinical studies and reviews link poststroke sleep quality, sleep duration, sleep disturbance, or sleep related symptoms with cognitive performance, PSCI, rehabilitation participation, and functional outcome.	Establishes the clinical overlap and relevance of sleep cognition interactions after stroke, particularly for attention and executive related outcomes.	Direct clinical evidence	Mostly observational or cross sectional evidence; sleep measures and cognitive outcomes are heterogeneous; causal direction remains uncertain.
Sleep phenotype heterogeneity and mechanisms	Chen et al. (2024), Zhang et al. (2026), Pawluk et al. (2025), Rissardo et al. (2025), Lv et al. (2023), Pérez-Carbonell and Bashir (2020), Mayer et al. (2011), Baillieul et al. (2023), Cai et al. (2021), and Sateia (2014)	PSSD includes insomnia, sleep fragmentation, OSA, hypersomnolence, and circadian disruption, with mechanisms such as hyperarousal, intermittent hypoxia, inflammation, and circadian misalignment.	Supports separating sleep phenotypes when testing network level and executive function outcomes.	Clinical and mechanistic context	Sleep phenotypes are often grouped together; objective sleep measures are inconsistently used.
PSCI and large scale network vulnerability	Siegel et al. (2016), Grefkes and Fink (2014), Wang et al. (2024), Menon (2011), Ruan et al. (2025), Carrera and Tononi (2014), Salvalaggio et al. (2020), Koh et al. (2021), Cole et al. (2013), Dosenbach et al. (2008), Yue et al. (2023), Anticevic et al. (2012), Sagues et al. (2025), Kolskår et al. (2022), Schimmelpfennig et al. (2023), Uddin (2015), Varjačić et al. (2018), Bonkhoff et al. (2021a, 2021b), and Frontzkowski et al. (2024)	Poststroke cognitive impairment is associated with focal lesion effects, diaschisis, structural disconnection, and altered static, dynamic, or effective connectivity involving the DMN, FPN, and SN.	Defines the vulnerable network substrate on which sleep disturbance may impose additional functional stress.	Direct stroke network evidence, not sleep specific	Sleep phenotypes are rarely measured; lesion location, stroke stage, cognitive domains, and imaging methods vary substantially.
Sleep disturbance and large scale networks outside stroke	De Havas et al. (2012), Chee and Zhou (2019), Ben Simon et al. (2017), Goulden et al. (2014), Pesoli et al. (2022), Tononi and Cirelli (2014), Xu et al. (2023), Zhang et al. (2025), Farahani et al. (2019), Dai et al. (2020), Qi et al. (2021), Wei et al. (2020), Prilipko et al. (2011), Marques et al. (2018), Khazaie et al. (2017), Schiel et al. (2020), Sämann et al. (2011), and Zheng et al. (2023)	Sleep deprivation, sleep restriction, insomnia, OSA, and sleep fragmentation are associated with altered DMN, salience, frontoparietal, and attention network connectivity, reduced network segregation, and impaired executive performance.	Provides biological plausibility that disturbed sleep can perturb network flexibility required for executive control.	Indirect mechanistic evidence	Most evidence comes from non stroke populations; experimental sleep loss may not fully model chronic poststroke sleep disturbance.
Salience network switching and executive control	Seeley et al. (2007), Sridharan et al. (2008), Goulden et al. (2014), Anticevic et al. (2012), Schimmelpfennig et al. (2023), Uddin (2015), Varjačić et al. (2018), Wei et al. (2020), Brown et al. (2019), and Spreng et al. (2010)	SN, fronto insular, and cingulate systems support salience detection and transitions between default mode and executive control states; altered SN executive connectivity has been linked to cognition and insomnia.	Supports SN mediated switching as the candidate bridge linking sleep disturbance with DMN/FPN dyscoordination and executive dysfunction.	Mechanistic bridge evidence	The poststroke sleep SN-DMN-FPN executive pathway has not been directly validated as a full causal chain.
Stroke specific bridge evidence and intervention testability	Cohen (2018), Hutchison et al. (2013), Ruan et al. (2025), Friston et al. (1997), Elton and Gao (2014), Bonkhoff et al. (2021a, 2021b), Baillieul et al. (2023), Cai et al. (2021), Wang et al. (2022), Zhu et al. (2023), Liu et al. (2023), Smith et al. (2018), and Perrotta et al. (2021)	Stroke specific sleep reviews and imaging studies suggest sleep related brain dysfunction, while dynamic/effective connectivity, task imaging, actigraphy, PSG, and intervention studies provide tools to test the pathway.	Identifies feasible methods to examine temporal ordering, mediation, moderation, and treatment responsiveness.	Preliminary stroke specific and translational evidence	Few cohorts combine objective sleep assessment, network level imaging, executive tasks, and longitudinal or intervention based designs.

This table stratifies the evidence supporting the proposed framework by proximity to the central sleep network executive hypothesis. The categories distinguish direct clinical evidence, stroke specific network evidence, indirect mechanistic evidence, preliminary bridge evidence, and translational evidence.

This stratification highlights an important asymmetry in the current literature. The clinical association between poststroke sleep disturbance and cognitive impairment is relatively well supported, and triple network abnormalities in poststroke cognitive impairment have been increasingly reported. Non stroke sleep research also provides biological plausibility that sleep disruption can alter large scale network dynamics. Direct evidence for the proposed pathway remains limited. No stroke cohort has yet tested whether objectively measured sleep phenotypes are linked to altered SN-DMN-FPN dynamics and domain specific executive impairment within a longitudinal or interventional design. The framework should therefore be viewed as a testable mechanistic hypothesis rather than an established causal pathway.

## 7 A testable mechanistic framework

The account advanced here is interactional rather than linear. Stroke leaves a structurally vulnerable network architecture, and disturbed sleep imposes an additional functional stress on that system. Executive dysfunction may become apparent when this combined burden exceeds the brain’s capacity to maintain flexible transitions between internally oriented and task directed states. Figure 3 summarizes this proposed sleep network executive pathway and distinguishes established clinical or network associations from hypothesized mechanisms requiring direct validation.

**Figure 3:**
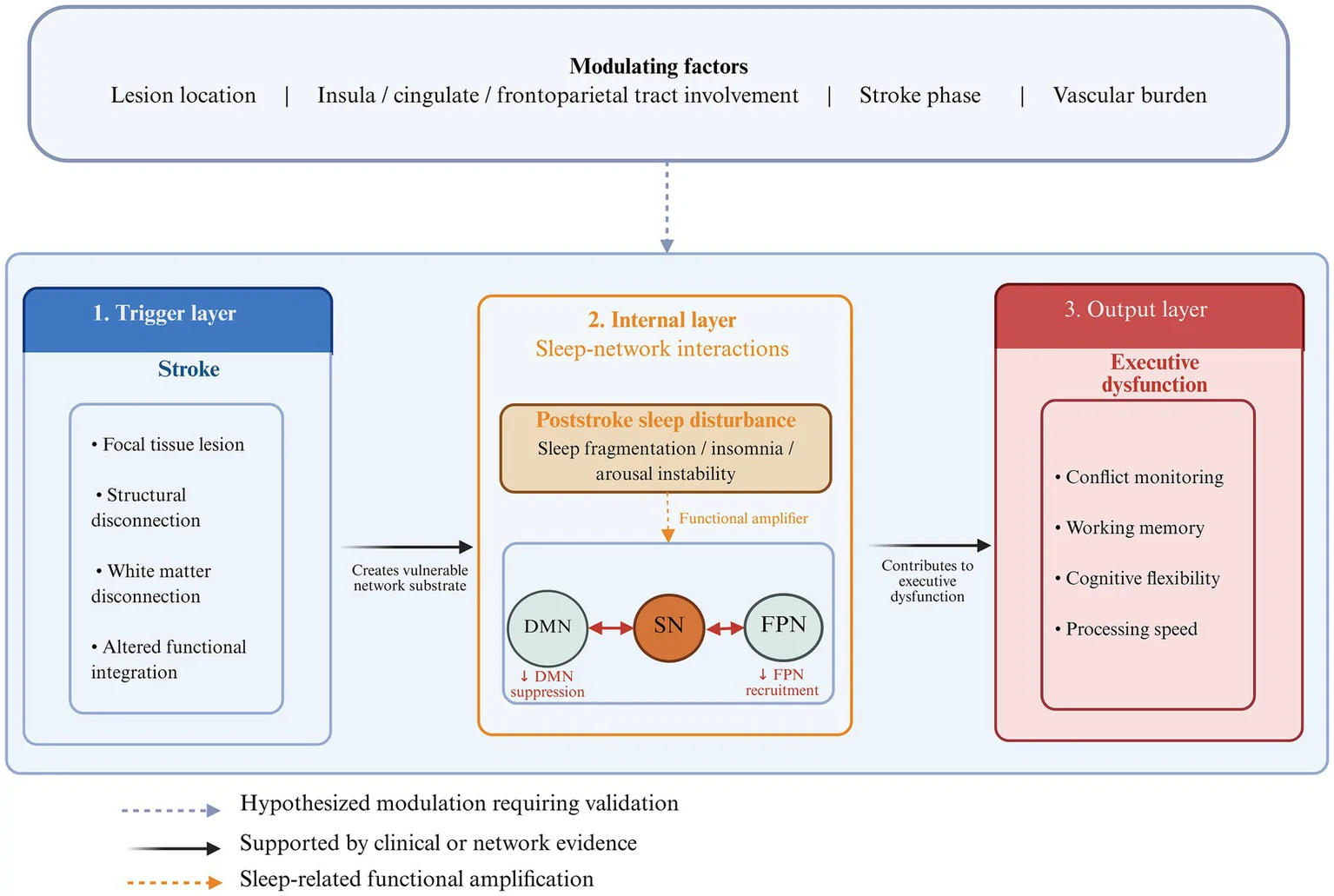
Proposed sleep network executive pathway after stroke. Stroke related lesions and disconnection may create a vulnerable network substrate, while selected sleep phenotypes may act as functional amplifiers of impaired SN-DMN-FPN coordination, contributing to executive dysfunction.

Stroke-related injury may create the initial vulnerability through focal tissue damage, diaschisis, and disruption of long-range structural connections (Carrera and Tononi, 2014; Salvalaggio et al., 2020). Sleep fragmentation, insomnia, and arousal dysregulation may then amplify the functional consequences of an already compromised system (Wei et al., 2020; Zheng et al., 2023). This remains a hypothesis: experimental fragmentation does not invariably impair cognition (Ferri et al., 2010), and the additional vulnerability conferred by stroke requires direct testing. Within this model, the SN is a plausible candidate mediator. Its role in detecting salient signals and coordinating transitions between the DMN and FPN (Seeley et al., 2007; Sridharan et al., 2008; Goulden et al., 2014; Wei et al., 2020) makes altered SN function a candidate link between sleep disturbance and executive performance. Inefficient switching could leave DMN activity insufficiently suppressed during task demands while delaying or weakening FPN recruitment. This would increase interference from internally oriented processing and reduce the availability of executive control resources (Anticevic et al., 2012; Brown et al., 2019; Spreng et al., 2010). The SN is proposed as one contributing mechanism; it need not explain every sleep-related cognitive effect.

The model predicts that greater sleep fragmentation or insomnia severity will be associated with altered SN-centered dynamics, such as longer dwell time in prespecified DMN-dominant states or fewer transitions toward task-relevant control states (Hutchison et al., 2013; Wei et al., 2020). Lesions or disconnection involving fronto-insular, cingulate, or frontoparietal pathways are proposed modifiers of this association (Kolskår et al., 2022; Varjačić et al., 2018; Frontzkowski et al., 2024; Kumral et al., 2019). SN-mediated switching inefficiency should account for a meaningful part of the sleep-executive association. Sleep or neuromodulatory interventions provide opportunities to test this prediction using domain-specific outcomes (Wilckens et al., 2014; Zhu et al., 2023; Liu et al., 2023), although a treatment benefit alone would not establish mediation.

### 7.1 Findings that would refute the proposed pathway

The framework proposes that altered SN-DMN-FPN coordination partly mediates the relationship between disturbed sleep and executive dysfunction after stroke. A fair test requires a defined population, reliable measures, and prespecified expectations about the direction, timing, and minimum size of the effects. Three patterns would argue against this account:

- Objectively measured sleep disruption is associated with executive impairment, but has no meaningful association with the prespecified network measure. This would challenge the proposed link between sleep disturbance and network dysfunction.
- A sleep intervention produces sustained improvements in sleep and executive function, yet neither the predicted network change nor a meaningful indirect effect is present. The proposed pathway would then fail to explain recovery in that population.
- Sleep improvement is accompanied by the predicted network changes, but these changes do not predict later executive gains, and the indirect effect is negligible. This would challenge the network-to-executive link.

These findings would provide evidence against the pathway only if the estimates were precise enough to rule out effects considered meaningful. Equivalence testing offers one way to make this distinction using prespecified bounds (Lakens, 2017). A nonsignificant result with a wide confidence interval remains inconclusive. Likewise, an intervention that fails to improve sleep, or measurements taken outside the expected time course, would provide a weak test. Replicated findings that exclude meaningful mediation would require revision or rejection of the proposed pathway in the population studied, even if the broader association between sleep and cognition remained intact.

### 7.2 Eligibility and confounder control

An initial study could focus on medically stable adults with imaging-confirmed ischemic stroke assessed within a defined recovery period, such as 3–12 months. This window is a practical design choice, not an established eligibility requirement. Active delirium or medical instability may warrant deferred assessment. Language, visual, and motor impairments should be accommodated wherever possible; exclusion is justified when primary measurements remain uninterpretable despite these adaptations. MRI contraindications would affect eligibility for imaging rather than necessarily for the entire study. Untreated severe OSA requires particular attention in studies of insomnia or sleep fragmentation. Patients with an apnea–hypopnea index ≥30 events/h could enter the primary analysis after treatment or form a separate respiratory subgroup (Lv et al., 2023). In an OSA-focused study, these patients would instead be central to the investigation, with hypoxic burden, treatment status, and adherence documented. Questionnaires alone cannot establish the diagnosis, and PSG is preferred for diagnostic assessment in patients with a history of stroke (Kapur et al., 2017). Insomnia and OSA may coexist and should not be treated as mutually exclusive categories. Stable use of centrally acting medication need not lead to exclusion. Drug class, dose, indication, dosing time, and recent changes are relevant to interpretation, while the required period of stability will depend on the drug. Necessary treatment should not be withdrawn for study participation. Other potential confounders include mood, pain, fatigue, vascular burden, premorbid cognition, rehabilitation exposure, and lesion characteristics. Their inclusion in statistical models should follow the assumed causal relationships. For example, mood changes after treatment may partly mediate recovery and should not automatically be adjusted away. Treatment randomization alone also does not eliminate confounding between network changes and executive outcomes. The primary comparison might distinguish insomnia or fragmentation without OSA, OSA alone, co-occurring disorders, and neither condition. Stroke stage and subtype should be recorded separately. A limited number of prespecified comparisons and lesion-based interactions would be more informative than dividing the sample into many small subgroups. Continuous measures can retain information where categorical grouping is unnecessary.

### 7.3 Power and sample size

No single sample size can be recommended for this framework. The main target is the indirect effect through network function, which may require a larger sample than detecting a sleep–cognition association alone. Monte Carlo simulations can guide planning for the intended longitudinal model (Thoemmes et al., 2010). Relevant inputs include plausible effects along both paths, measurement reliability, within-person correlations, and losses from attrition or imaging quality control. Site differences and planned interactions may further increase requirements. Planning should address both the desired power, commonly 80% or 90%, and the precision needed to rule out meaningful effects in equivalence tests (Lakens, 2017). Pilot data can inform feasibility and reliability, but their effect estimates alone provide an uncertain basis for sample size calculations. Repeated imaging windows do not substitute for independent participants. Limiting confirmatory endpoints, accounting for multiple testing, and seeking independent replication would strengthen the resulting evidence. Table 2 summarizes the proposed measures and study designs for testing the sleep–network–executive pathway after stroke.

**Table 2:** Candidate operationalization of the proposed sleep-network-executive framework after stroke.

Component to test	Suggested measures	Findings relevant to the hypothesis	Key design considerations
Sleep phenotype	Pre-specify the main sleep exposure, such as sleep continuity, insomnia severity, or respiratory disturbance; combine subjective and objective measures where feasible (Rissardo et al., 2025; Baillieul et al., 2023; Smith et al., 2018).	The proposed pathway predicts that the selected sleep exposure is associated with altered network coordination.	Distinguish insomnia/fragmentation, OSA, and their co-occurrence. Document OSA treatment and adherence; define how untreated severe OSA will be handled (Kapur et al., 2017).
Network vulnerability after stroke	Assess lesion location and volume, structural disconnection, and baseline network connectivity (Siegel et al., 2016; Grefkes and Fink, 2014; Wang et al., 2024; Kolskår et al., 2022; Frontzkowski et al., 2024).	A stronger sleep–executive association in patients with greater network disruption would support the proposed vulnerability effect.	Prespecify a limited number of lesion-by-sleep interactions. Record stroke stage and subtype, vascular burden, medication exposure, mood, and rehabilitation.
SN–DMN–FPN coordination	Select a primary network measure using dynamic or effective connectivity, supplemented by task-based imaging where appropriate (Cohen, 2018; Hutchison et al., 2013; Ruan et al., 2025; Friston et al., 1997; Elton and Gao, 2014).	Sleep-related differences in coordination should relate to executive performance. Precisely estimated negligible associations would challenge the relevant link.	Define the expected direction and measurement window. Assess reliability, head motion, and vigilance; resting-state variability alone does not establish impaired switching.
Executive demand	Prespecify one primary executive outcome and, where feasible, examine performance across task loads (Periáñez et al., 2021; Owen et al., 2005; Elton and Gao, 2014).	The model predicts more pronounced sleep-related difficulty when tasks require greater executive control.	Account for language, vision, motor speed, fatigue, and practice effects. Coordinate behavioral and imaging assessments.
Temporal ordering	Repeat sleep, network, and executive measurements, with assessment intervals chosen to capture the proposed sequence.	Sleep disturbance preceding network change, followed by executive decline or slower recovery, would support temporal ordering.	Adjust for baseline performance and prespecified confounders. Concurrent changes alone cannot establish the proposed sequence.
Response to intervention	Confirm improvement in the targeted sleep phenotype, then assess network change and later executive outcomes (Zhu et al., 2023; Liu et al., 2023).	Executive improvement despite precisely estimated negligible network change and indirect effects would challenge mediation through the proposed pathway.	Record adherence and concurrent treatment. Prespecify meaningful-effect bounds and consider alternative pathways; treatment efficacy alone does not establish network mediation (Lakens, 2017).
Power and precision	Use simulation to plan sample size for the primary longitudinal indirect effect (Thoemmes et al., 2010).	Estimates must distinguish a meaningful mediated effect from an effect small enough to challenge the hypothesis.	Account for measurement reliability, attrition, imaging exclusions, and site effects. Plan interaction analyses separately and limit confirmatory endpoints.

These are proposed research options, not validated diagnostic criteria. Expected findings are hypotheses to be tested. A nonsignificant result alone does not refute the pathway; contradictory evidence requires reliable measures and sufficient precision to exclude prespecified meaningful effects (Lakens, 2017). Routine clinical assessment is summarized in Table 3.

### 7.4 Applicability across stroke subtypes

Evidence for the framework comes mainly from ischemic stroke cohorts (Wang et al., 2024; Li et al., 2019; Wang et al., 2022), and its applicability to hemorrhagic stroke remains uncertain. Intracerebral hemorrhage also disrupts brain networks, but mass effect, perihematomal edema, and injury from blood breakdown products may alter the time course of network dysfunction (Greenberg et al., 2022). These differences could affect how sleep disturbance relates to executive recovery, although this has not been directly tested. Initial validation could focus on ischemic stroke, with separate testing in intracerebral hemorrhage. Studies enrolling both subtypes should prespecify how their effects will be compared. Subarachnoid hemorrhage warrants separate consideration rather than being grouped with intracerebral hemorrhage.

## 8 Clinical implications, limitations, and future directions

The clinical value of this framework depends on whether it helps identify which stroke survivors are vulnerable and which sleep problems deserve further assessment. At present, it can guide assessment and research questions, but it does not justify selecting treatment from a network scan. Insomnia, fragmented sleep, OSA, daytime sleepiness, and circadian disruption should be described separately because they may have different cognitive consequences. A global sleep complaint or fatigue score does not identify its cause. History from the patient and, where appropriate, a caregiver can guide focused assessment; actigraphy may help characterize sleep continuity and rest-activity patterns (Smith et al., 2018), while suspected OSA warrants diagnostic evaluation (Kapur et al., 2017). Table 3 summarizes assessments that can be used in current clinical settings without assuming that the proposed mechanism has been established.

**Table 3:** Practical considerations for sleep and executive assessment after stroke.

Clinical focus	Suggested assessment	Clinical use and interpretation
Insomnia and fragmented sleep	Ask about difficulty falling asleep, repeated awakenings, early waking, and daytime consequences. The PSQI can summarize subjective sleep complaints; a sleep diary can clarify their pattern.	Use the findings to guide further sleep assessment and identify complaints to follow over time. Actigraphy may help when sleep continuity or timing is unclear, although reduced movement due to paresis can complicate interpretation. A PSQI score alone does not establish a diagnosis.
Possible sleep apnea	Ask about snoring, witnessed breathing pauses, gasping, and daytime sleepiness. Review previous OSA investigations, treatment, and adherence.	Suspected OSA warrants diagnostic evaluation; symptoms and screening scores cannot confirm or exclude it. Absence of sleepiness does not rule out OSA. PSG is preferred for diagnostic assessment in patients with a history of stroke.
Sleep timing and daytime alertness	Record bedtimes, wake times, naps, light exposure, and variation across days. Distinguish sleepiness from fatigue, and review pain, mood, and medication timing.	A diary or actigraphy can help characterize irregular sleep patterns and guide further assessment. Potential contributors to reduced alertness should be considered before attributing poor cognitive performance to network dysfunction.
Executive function	Select measures according to the clinical concern: Inhibitory control: Stroop interference. Cognitive switching: Trail Making Test Part B, with B − A considered where appropriate. Working memory: Digit Span backward.	Use domain-specific assessment to characterize difficulties that global cognitive screening may miss and to guide follow-up. These tasks draw on several abilities; results require consideration of language, vision, motor speed, education, and fatigue. Where standard testing is unsuitable, a validated stroke-adapted task may be preferable.
Follow-up	Repeat relevant sleep and executive measures under comparable conditions. Document treatment, medication changes, and rehabilitation exposure.	Assess whether changes in test performance are accompanied by improvements in everyday function. Practice effects and changes in testing conditions should be considered when interpreting repeated assessments.

This table supports clinical characterization, referral, and follow-up; it is not a validated assessment battery for the proposed pathway. SN–DMN–FPN imaging is not a prerequisite for routine assessment. Parallel improvements in sleep and executive function do not, by themselves, establish network mediation.

The framework should be interpreted in light of several limitations. This review was designed as a targeted narrative synthesis rather than a systematic review or meta analysis. Although the search strategy, evidence tiers, and SANRA based self audit were specified, formal study level risk of bias assessment was not applied to all primary studies. The evidence base is methodologically heterogeneous, with many observational and cross sectional studies, variable definitions of poststroke sleep disturbance, and inconsistent use of objective sleep measures. Cognitive outcomes are also unevenly characterized, with many studies relying on global screening tools rather than executive tasks sensitive to switching inefficiency. Neuroimaging findings are difficult to compare across studies because of differences in stroke stage, lesion location, imaging modality, preprocessing strategy, and analytic approach. Several mechanistic inferences also rely on non-stroke sleep research, which provides biological plausibility but cannot directly establish the proposed pathway in stroke cohorts. Because study designs and outcome metrics were not sufficiently commensurate, quantitative pooling was not performed. The conclusions should therefore be read as a structured conceptual synthesis, not as evidence that the proposed causal pathway has already been validated.

Prospective validation should combine repeated sleep phenotyping, network imaging, and executive assessment in the same individuals. Figure 4 outlines this design; the operational choices in Table 2 and the falsification criteria above specify what would count as support or contradiction. Intervention studies should establish whether sleep improves before testing whether network change precedes executive recovery. They should also compare alternative explanations, including mood, daytime activity, hypoxia, and rehabilitation exposure. A treatment can be clinically useful even when its benefit is not mediated by the proposed network pathway.

**Figure 4:**
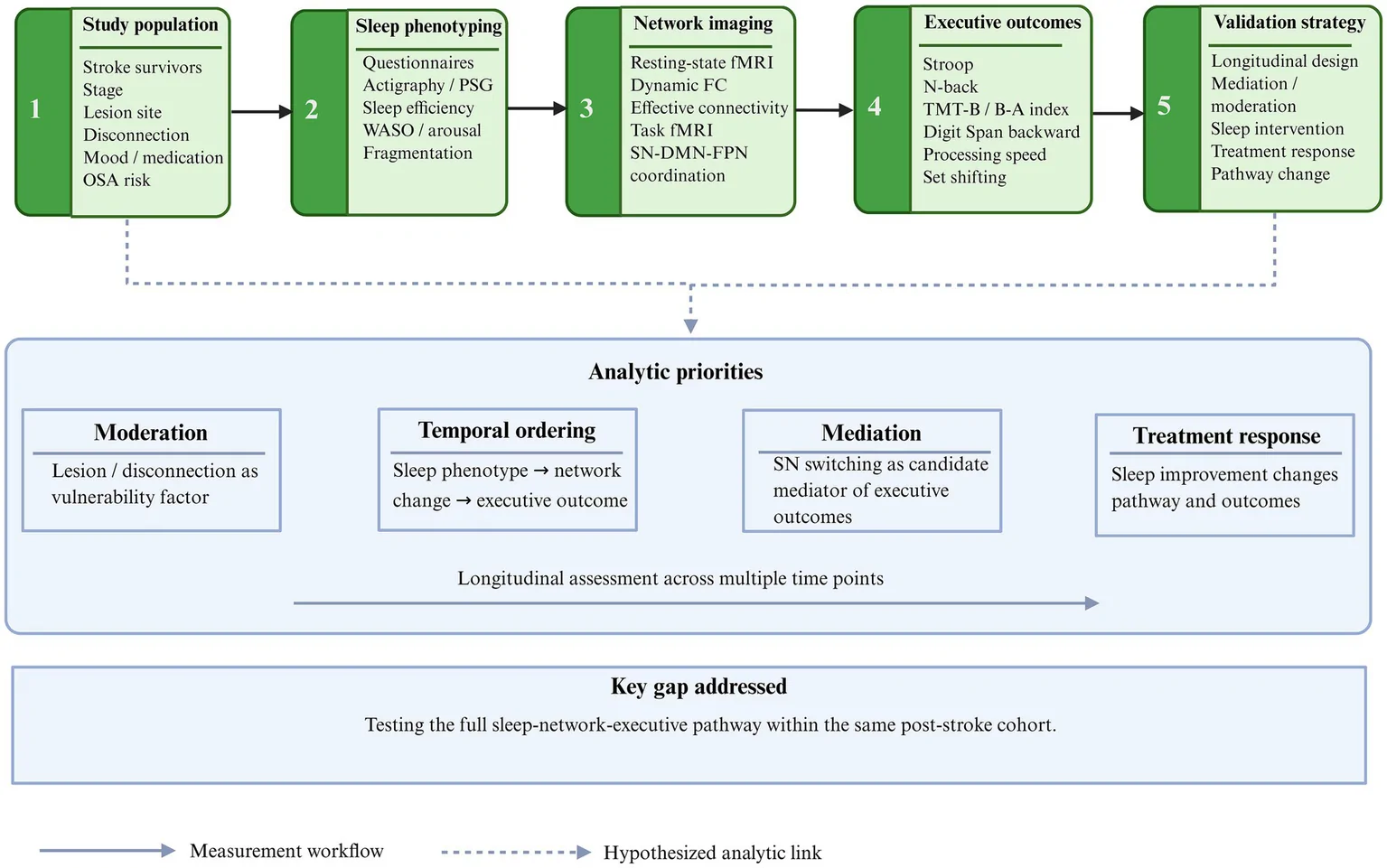
Multimodal validation framework for testing the sleep-network-executive hypothesis after stroke. The framework aligns study population characterization, objective sleep phenotyping, network imaging, executive outcomes, and longitudinal or interventional validation within a single stroke cohort. Prespecified analyses should test temporal ordering, mediation by SN-DMN-FPN coordination, and a limited set of lesion-related modifiers. Table 2 provides operational design choices; Section 8 defines contradictory findings and power considerations. Parallel sleep and executive improvement alone does not establish network mediation.

The framework provides a way to ask which sleep phenotypes are associated with executive difficulty and whether network changes help explain that association. Its value will depend on prospective tests that can distinguish the proposed mechanism from alternative pathways and reject it when the predicted relationships are absent.

## 9 Conclusion

Sleep disturbance and executive dysfunction are both common after stroke and carry clear clinical consequences, but their relationship is not fully explained by symptom based or lesion centered accounts. We propose that poststroke sleep disturbance, particularly sleep fragmentation, insomnia, and related arousal dysregulation, may contribute to executive dysfunction by disrupting large scale network coordination.

In this model, disturbed sleep further destabilizes a vulnerable poststroke network architecture by impairing salience network mediated switching between default mode and frontoparietal systems. Sleep disturbance is therefore not viewed simply as a parallel comorbidity, but as a potential functional stressor that may interact with structural and network level vulnerability to shape executive performance. The proposed sleep network executive pathway remains a testable hypothesis rather than an established mechanism. Current evidence is constrained by heterogeneous sleep phenotyping, frequent reliance on global cognitive outcomes, and the limited number of stroke studies combining objective sleep assessment with dynamic or directional network measures and domain specific executive tasks.

This review supports a shift toward a network level understanding of poststroke cognitive dysfunction and highlights sleep disturbance as a potentially modifiable contributor to impaired recovery. Clarifying this relationship may guide more precise patient stratification, mechanism sensitive outcome selection, and future rehabilitation oriented intervention studies.

## Data Availability

The original contributions presented in the study are included in the article/Supplementary material, further inquiries can be directed to the corresponding authors.
