# Supplementary material for: Poststroke sleep phenotypes and executive dysfunction: toward a sleep-network-executive framework
Source: Front Neurosci. 2026 Oct 21;20:1978796. doi: 10.3389/fnins.2026.1978796 (PMC13731308; doi:10.3389/fnins.2026.1978796)
Supplement: Supplementary file 1 [file Supplementary_file_1.DOCX]

Supplementary Tables

Supplementary material for the targeted narrative synthesis: post-stroke sleep phenotypes and executive dysfunction.

# Supplementary Table S1. Database-specific search strategies

The review was designed as a targeted narrative synthesis rather than a systematic review or meta-analysis. Searches covered literature available up to June 19, 2026

| **Database / source** | **Coverage and date** | **Search strategy** | **Limits / notes** |
| --- | --- | --- | --- |
| PubMed | Database inception to June 19, 2026 | ("Stroke"[Mesh] OR stroke*[tiab] OR poststroke[tiab] OR "post-stroke"[tiab] OR "ischemic stroke"[tiab] OR "ischaemic stroke"[tiab] OR "cerebral infarction"[tiab] OR "cerebrovascular accident*"[tiab]) AND ("Sleep Wake Disorders"[Mesh] OR "Sleep"[Mesh] OR "Sleep Apnea, Obstructive"[Mesh] OR insomnia[tiab] OR "sleep disturbance*"[tiab] OR "sleep disorder*"[tiab] OR "sleep fragmentation"[tiab] OR "sleep quality"[tiab] OR "obstructive sleep apnea"[tiab] OR "obstructive sleep apnoea"[tiab] OR OSA[tiab] OR hypersomnolence[tiab] OR hypersomnia[tiab] OR "circadian rhythm*"[tiab] OR chronotype[tiab]) AND (("Cognition Disorders"[Mesh] OR "Executive Function"[Mesh] OR "Attention"[Mesh] OR "Memory, Short-Term"[Mesh] OR "post-stroke cognitive impairment"[tiab] OR PSCI[tiab] OR "cognitive impairment"[tiab] OR "executive dysfunction"[tiab] OR "executive function*"[tiab] OR attention[tiab] OR "working memory"[tiab]) OR ("Magnetic Resonance Imaging"[Mesh] OR "Brain Mapping"[Mesh] OR "default mode network"[tiab] OR DMN[tiab] OR "frontoparietal network"[tiab] OR FPN[tiab] OR "central executive network"[tiab] OR CEN[tiab] OR "salience network"[tiab] OR "large-scale network*"[tiab] OR "brain network*"[tiab] OR "functional connectivity"[tiab] OR "dynamic functional connectivity"[tiab] OR "effective connectivity"[tiab] OR fMRI[tiab] OR "resting-state"[tiab] OR "rs-fMRI"[tiab])) | Peer-reviewed original studies and relevant reviews were prioritized. No formal pooled-effect search filter was applied. |
| Web of Science Core Collection | Database inception to June 19, 2026 | TS=((stroke* OR poststroke OR "post-stroke" OR "ischemic stroke" OR "ischaemic stroke" OR "cerebral infarction" OR "cerebrovascular accident*") AND (insomnia OR "sleep disturbance*" OR "sleep disorder*" OR "sleep fragmentation" OR "sleep quality" OR "obstructive sleep apnea" OR "obstructive sleep apnoea" OR OSA OR hypersomnolence OR hypersomnia OR "circadian rhythm*" OR chronotype) AND (("post-stroke cognitive impairment" OR PSCI OR "cognitive impairment" OR "executive dysfunction" OR "executive function*" OR attention OR "working memory") OR ("default mode network" OR DMN OR "frontoparietal network" OR FPN OR "central executive network" OR CEN OR "salience network" OR "large-scale network*" OR "brain network*" OR "functional connectivity" OR "dynamic functional connectivity" OR "effective connectivity" OR fMRI OR "resting-state" OR "rs-fMRI"))) | Used for citation coverage across clinical, sleep, cognitive neuroscience, and neuroimaging literature. |
| Scopus | Database inception to June 19, 2026 | TITLE-ABS-KEY((stroke* OR poststroke OR "post-stroke" OR "ischemic stroke" OR "ischaemic stroke" OR "cerebral infarction" OR "cerebrovascular accident*") AND (insomnia OR "sleep disturbance*" OR "sleep disorder*" OR "sleep fragmentation" OR "sleep quality" OR "obstructive sleep apnea" OR "obstructive sleep apnoea" OR OSA OR hypersomnolence OR hypersomnia OR "circadian rhythm*" OR chronotype) AND (("post-stroke cognitive impairment" OR PSCI OR "cognitive impairment" OR "executive dysfunction" OR "executive function*" OR attention OR "working memory") OR ("default mode network" OR DMN OR "frontoparietal network" OR FPN OR "central executive network" OR CEN OR "salience network" OR "large-scale network*" OR "brain network*" OR "functional connectivity" OR "dynamic functional connectivity" OR "effective connectivity" OR fMRI OR "resting-state" OR "rs-fMRI"))) | Used to capture interdisciplinary sources across sleep medicine, rehabilitation, neurology, and network neuroscience. |
| Google Scholar and manual citation screening | Searched up to June 19, 2026 | "post-stroke" "sleep disturbance" "executive function"; "stroke" "insomnia" "cognitive impairment" "functional connectivity"; "post-stroke cognitive impairment" "default mode network" "frontoparietal network" "salience network"; "sleep fragmentation" "default mode network" "executive function"; "obstructive sleep apnea" "stroke" "cognitive impairment" "functional connectivity" | Used as supplementary citation tracking because Google Scholar does not support fully reproducible complex Boolean searches. Reference lists of key reviews and primary studies were manually screened. |

# Supplementary Table S2. Evidence-tier definitions and mapping criteria

Evidence was mapped according to proximity to the proposed sleep-network-executive pathway.

| **Evidence tier** | **Operational definition** | **Mapping criteria** | **Role in framework development** |
| --- | --- | --- | --- |
| Direct clinical evidence | Stroke cohort studies linking post-stroke sleep disturbance or specific sleep phenotypes with cognitive or executive outcomes. | Mapped to this tier when a stroke cohort reported both sleep measures and cognitive or executive outcomes. | Establishes the clinical relevance of sleep-cognition associations after stroke. |
| Stroke specific network evidence | Stroke studies reporting DMN, FPN, SN, dynamic connectivity, effective connectivity, structural disconnection, diaschisis, or lesion-network vulnerability related to cognition. | Mapped to this tier when neuroimaging or network measures were obtained in stroke or PSCI cohorts, regardless of whether sleep was assessed. | Defines the vulnerable post-stroke network substrate on which sleep-related perturbation may act. |
| Preliminary bridge evidence | Studies partially linking post-stroke sleep phenotypes with neuroimaging, regional brain dysfunction, network organization, or cognitive outcomes. | Mapped to this tier when sleep-related measures and stroke-related brain or cognitive outcomes were both present, but the complete SN-DMN-FPN-executive pathway was not tested. | Provides partial connection between sleep phenotypes, brain function, and cognitive vulnerability after stroke. |
| Indirect mechanistic evidence | Non-stroke studies of sleep deprivation, sleep restriction, insomnia, OSA, circadian disruption, or sleep fragmentation reporting network or executive-function effects. | Mapped to this tier when findings informed DMN, FPN, SN, network segregation, task-state reconfiguration, arousal instability, or executive performance. | Provides biological plausibility that disturbed sleep can alter network flexibility and executive control. |
| Translational evidence | Sleep-focused, respiratory, circadian, behavioral, or neuromodulatory intervention studies relevant to executive recovery or network modulation. | Mapped to this tier when an intervention plausibly targeted sleep continuity, arousal, respiration, circadian regulation, network function, or executive outcomes. | Identifies how the proposed pathway could be tested for modifiability and treatment responsiveness. |
| Not retained for framework synthesis | Sources without clear empirical, methodological, or conceptual relevance to stroke, sleep, cognition, or large-scale network function. | Not retained when no prespecified evidence tier was addressed. Studies were not excluded because findings were negative, inconsistent, or contrary to the proposed framework. | Reduces irrelevant citation while preserving relevant contradictory or null evidence. |

# Supplementary Table S3. SANRA-based self-audit

SANRA items were used as a narrative-review transparency check. Scores are self-audit ratings from 0 to 2 for each item.

| **SANRA item** | **Score** | **How addressed in this review** |
| --- | --- | --- |
| **Justification of the article's importance for the readership** | 2 | The review addresses the clinically frequent overlap between post-stroke sleep phenotypes and executive dysfunction and frames it as a rehabilitation-relevant brain-behavior problem. |
| **Statement of concrete aims or formulation of questions** | 2 | The review explicitly aims to develop a testable sleep-network-executive framework rather than summarize associations or estimate pooled effects. |
| **Description of the literature search** | 1 | Databases, final search date, search concepts, supplementary citation tracking, and evidence-mapping logic are reported. Full database-specific search strings are provided in Supplementary Table S1. |
| **Referencing** | 2 | Key claims are linked to distinct evidence layers, including clinical, stroke-network, sleep-network, bridge, and translational evidence. |
| **Scientific reasoning** | 2 | Evidence is separated according to proximity to the proposed pathway, and hypothesized mechanisms are distinguished from established associations. |
| **Appropriate presentation of data** | 2 | Tables and figures summarize evidence proximity, framework components, pathway logic, and future validation strategies. |
| **Overall self-audit interpretation** | 11/12 | The review meets most SANRA criteria for a structured narrative/conceptual review. The search is transparent and reproducible but not intended to meet PRISMA standards for systematic reviews. |
